# Spillover Effects of Paid Functions on Physicians’ Unpaid Knowledge Activities: Quasi-Experimental Approach

**DOI:** 10.2196/58688

**Published:** 2024-12-10

**Authors:** Xuan Liu, Xiaotong Chi, Ming Chen, Wen Sun, Jia Li

**Affiliations:** 1 East China University of Science and Technology Shanghai China

**Keywords:** health knowledge contribution, economic incentives, diversity, propensity score matching, multi-period difference in differences

## Abstract

**Background:**

To promote sustained contributions by physicians to online health care communities, these platforms have introduced a content payment model that offers economic incentives for physicians’ online knowledge activities. However, the impact of these paid features on unpaid knowledge activities remains unexplored.

**Objective:**

This study investigated how the introduction of economic incentives in online medical communities affects physicians’ unpaid knowledge activities in the community.

**Methods:**

The data for this study were obtained from the Haodf Online platform in China, which has implemented paid scenarios for its science popularization function, providing economic benefits to physicians. The dataset, which comprises panel data, includes 7453 physicians who participated in both paid and unpaid knowledge contributions on the website. This study examined the impact of paid knowledge activities on physicians’ free knowledge contributions, focusing on dimensions including knowledge quantity, quality, and diversity. To address the timing discrepancies in physicians’ participation in paid activities, we used a quasi-experimental design that combined the approach of propensity score matching and multi-period difference in differences.

**Results:**

In the balance test results of the propensity score matching, the absolute values of the SDs of all matching variables were mostly <5% after matching, ensuring the accuracy of the results obtained from the difference in differences method. This study found that participation in paid knowledge activities had a positive spillover effect on physicians’ free knowledge contributions, which manifested in the increase in post quantity (473.1%; *P*<.001), article length (108%; *P*=.009), function word frequency (0.6%; *P*=.001), causal word frequency (0.2%; *P*<.001), and content information entropy (6.6%; *P*=.006). The paid function led to a decrease in the consistency between titles and content (–115.5%; *P*<.001).

**Conclusions:**

The findings of this study contribute to the existing literature on the impact of economic incentives in the medical context. For the platform, providing economic incentives to physicians can have positive significance in promoting the development of the platform’s knowledge ecosystem and can effectively encourage physicians to contribute to both paid and free knowledge activities. This study provides a valuable reference for the platform to introduce a paid knowledge model, which is beneficial to the sustainable development of the platform.

## Introduction

### Background

The prosperity of online medical communities depends on the sustained participation of physicians, especially their knowledge contribution to the community. To encourage physicians’ contributions, online medical communities have implemented a content-charging model to provide economic rewards for physicians’ online knowledge activities. The success of platforms with similar revenue models, such as Quora and Zhihu Live, demonstrates that economic incentives enhance users’ performance in paid knowledge activities [1].

**Figure 1:**
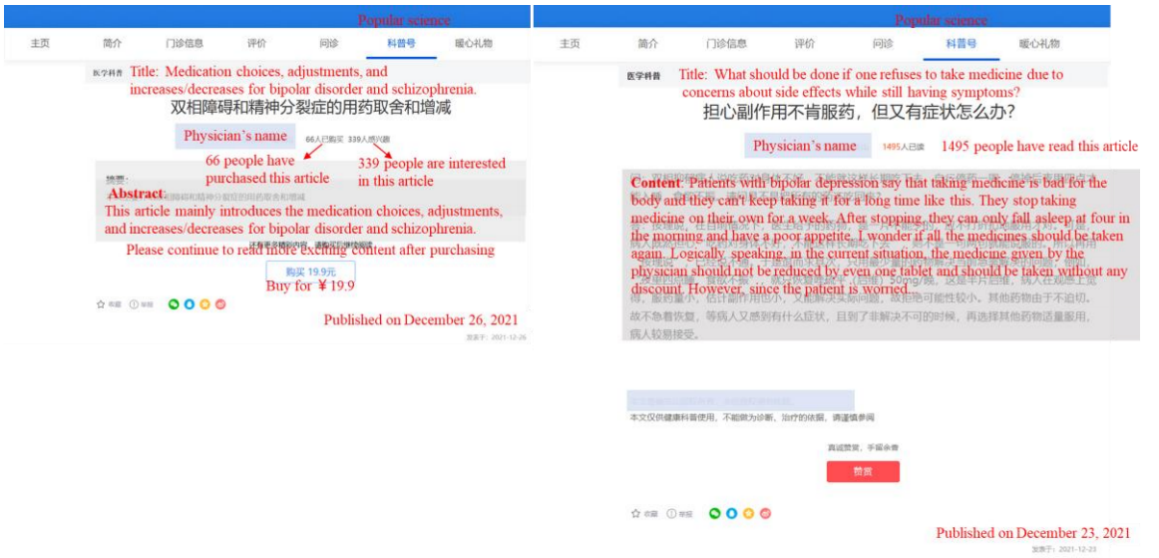
Example of physicians’ (A) paid and (B) unpaid knowledge contributions.

Providing incentives for physicians’ knowledge contributions in online medical communities is crucial for both physicians and patients. For physicians, offering channels to monetize their knowledge enhances their earnings from online contributions, increasing their willingness to share and efficiency in sharing knowledge. For patients, the availability of paid knowledge supplements their access to information, providing higher-quality and more specialized medical knowledge. Figure 1 illustrates the paid and free science popularization pages on the Haodf platform in China.

Incentive mechanisms are one of the most effective measures to encourage active participation in knowledge contribution within online communities. They hold significant value for the thriving development of online health communities. Effective incentive mechanisms can significantly enhance user engagement. Common online community incentives include economic incentives (such as monetary rewards and point systems) and social incentives (such as online reputation). This study focused on economic incentives, specifically manifested as patients paying fees to access paid articles published by physicians. The fees generated from this process directly translate into economic incentives for physicians. Thus, the core focus of this study was on the impact of economic incentives in the context of online health communities. Currently, paid knowledge and free knowledge coexist in online medical communities, and previous studies have found that economic incentives directly affect users’ knowledge contributions [2,3] and sustained participation [4]. However, it is also crucial to understand how economic incentives influence unpaid contributions to knowledge activities, which may affect overall community performance [5]. On the one hand, free content, which has no payment threshold and accumulates significantly, can reach a wider range of users and maintain user engagement on the platform. On the other hand, medical knowledge products are of public interest, and free medical knowledge provides a barrier-free channel for users to obtain medical information, effectively protecting the basic interests of patients. Therefore, if economic incentives result in physicians contributing less to free content, it would be detrimental to the long-term development of the community. Conversely, if economic incentives encourage physicians to further contribute to free content, it would significantly enhance physician-patient interactions within the community. Previous studies have confirmed that, in generalized knowledge communities, economic incentives have a spillover effect on nonincentive activities, boosting users’ overall participation in knowledge communities [5].

Although many scholars have conducted research in this field, several key issues are still unresolved. First, the findings from generalized knowledge communities may not apply to medical contexts. In online medical communities, physicians face significant pressures from offline diagnoses and clinical duties, limiting their time and energy for online knowledge services. Thus, the conclusions drawn from generalized knowledge communities may not be applicable. Second, there is no consensus on the spillover effects of economic incentives. Existing studies have not yet reached a unified conclusion on whether economic incentives have spillover effects on free service performance and the specific direction of the impact. The answer varies across different research contexts. In general, the impacts of participating in paid knowledge activities on contributions to free knowledge activities can be summarized as being both positive and negative. Positive impacts include (1) reputation establishment (engaging in free knowledge activities without thresholds can enhance reputation in the community and attract patients) [6], (2) community contribution (reciprocity motivates physicians to contribute to free knowledge activities due to the benefits received from paid activities) [7], and (3) exposure opportunity (the creation threshold of free knowledge is relatively low, and by sharing free knowledge, physicians can quickly obtain more exposure opportunities) [8]. Negative impacts include (1) the substitution effect (participation in paid knowledge activities leads to physicians’ inability to allocate sufficient time and energy for free knowledge activities) [9] and (2) the crowding-out effect (charging for knowledge activities weakens physicians’ intrinsic motivation to contribute freely, thus reducing the prosocial quality of physicians’ participation in free knowledge contribution) [10,11].

How does physicians’ participation in paid knowledge activities affect their contribution to free knowledge activities? For platforms, it is crucial to develop knowledge-paying models without harming the interests of the vast number of free knowledge-sharing groups and balance physicians’ free and paid knowledge contributions. This study examined the experience of a Chinese leading medical platform, Haodf Online. To clarify the differences in physicians’ free content contribution before and after economic incentives, this study used a quasi-experimental design that combines the propensity score matching (PSM) technique with the multi-period difference in differences (DID) analysis, and investigated on the spillover effect of paid knowledge services on physicians’ free knowledge services. The propensity score matching (PSM) method was used to construct comparable groups of paid and nonpaid physicians to solve the sample selection bias problem. The multi-period difference in differences (DID) method was used to compare the changes in physicians’ free content contribution before and after economic incentives. Its advantage lies in eliminating individual and time trends and accounting for policy stimuli across multiple time points. This research will contribute to the application of economic incentives in online medical communities.

### Literature Review

#### Physicians’ Online Knowledge Contribution

Research on online medical communities has emphasized the importance of knowledge services and knowledge activities. Scholars have extensively studied patient participation in these communities, finding that a primary motivation is to obtain informational support. Knowledge exchange and knowledge communication on the platform can improve the health status of patients. Knowledge exchange on these platforms provides answers with more quantity and more clinical significance than search engine results [12].

Nowadays, scholars have focused on the crucial role of physicians as professional service providers in promoting the prosperity of online medical communities. Current research primarily examines the factors and mechanisms influencing physicians’ knowledge contribution and the impact of knowledge exchange on service performance. Scholars have primarily studied the influencing factors and internal mechanisms behind physicians’ knowledge contribution while noting a trend from free sharing to paid knowledge exchange in online medical communities. Studies have found that both intrinsic and extrinsic motivations drive users to share knowledge voluntarily. Specifically, providing economic incentives for free question-and-answer services enhances users’ willingness to share their expertise [13]. Yang et al [14] discovered that both personal and social motivations positively influence physicians’ contributions to online health communities, with physicians’ professional status moderating this relationship. Some scholars have attempted to explore the connection between general free knowledge sharing and specific paid medical services by examining patients’ transition from seeking free information to engaging in paid consultations. For instance, Meng et al [15] confirmed the significant role played by reputation-establishing mechanisms in facilitating this transition. As the paid knowledge model in the medical field becomes commercialized, some scholars have started conducting more comprehensive research on payment models for medical expertise. For example, Qi et al [1] studied the external impact of physicians’ paid question-and-answer services, finding improvements in both the quantity and quality of their unpaid knowledge contributions. Regarding the influence of knowledge exchange between physicians and patients, establishing knowledge interaction significantly affects patients’ perceptions of medical services. Khurana et al [16] found that physicians’ active participation in responding to queries on online medical platforms can mitigate information asymmetry between physicians and patients, thereby increasing physician recommendations.

#### Spillover Effect of Incentives

Incentive mechanisms play a pivotal role in online communities, significantly influencing user engagement and contribution levels [17]. These mechanisms can be broadly categorized into intrinsic and extrinsic incentives. Intrinsic incentives arise from the internal satisfaction that users derive from participation, such as self-fulfillment [18], personal interests, knowledge sharing, and social interaction. Conversely, extrinsic incentives include material and social rewards. Material rewards motivate users through economic incentives, gift cards, and coupons. Social rewards encompass reputation [18], honors, ranking systems, and badges [19], enhancing users’ status and recognition within the community. Incentive mechanisms in online communities should be tailored to the community’s nature and user characteristics to promote sustainable and healthy development.

Recently, the impact of economic incentives on content contribution has garnered significant attention from scholars. Most previous question-and-answer platforms were free, focusing solely on free knowledge sharing. Therefore, compared with earlier free knowledge exchange platforms, the leading role of economic incentives in knowledge sharing remains to be further explored. Typically, economic incentives are used as a direct means to stimulate user content contribution. In previous studies, the primary forms of economic incentives have included direct economic rewards, coupons, product discounts, and online currency rewards.

In empirical studies, conclusions on the effects of economic incentives are not uniform. Some scholars believe that, in the short term, economic incentives on platforms have a positive effect, potentially increasing the number of comments [3,6]. Sun and Zhu [20] found that revenue from shared advertising increased the number and quality of blog posts, prompting bloggers to shift content to more popular topics. Regarding quality, economic incentives are believed to have no impact on performance [21], such as the quality of comments [3,6] or the quality of stock recommendations [22].

However, some studies have found that economic incentives are not effective and may even have a negative effect. Chen et al [23] found that price and tips had no significant effect on the length and quality of answers in question-and-answer communities. In addition, some scholars have found that economic incentives have a negative effect, with the introduction of economic incentives leading to a decrease in the number of online comments and the total contribution to the community [24].

Beyond examining how economic incentives directly affect paid activities, scholars have also focused on the interaction between incentive-based activities and other unpaid activities. There is no consensus on the spillover effect of economic incentives on free knowledge contributions. In the short term, economic incentives can enhance the quantity and quality of users’ free knowledge contributions [1,6] and increase users’ overall participation in the knowledge community but have no significant effect on users’ knowledge-seeking behavior [5]. In the long term, this effect is difficult to sustain [6]. Some studies have also found that economic incentives negatively affect users’ subsequent prosocial contributions, reducing their prosocial performance and efforts [25]. Regarding the impact of free knowledge sharing on paid knowledge activities, some scholars have found that free knowledge contributions can enhance user trust and promote user knowledge purchasing [15]. However, some scholars believe that, due to the limited time and energy of service providers, the spillover effect of free activities will turn negative after a certain point [26].

#### Research Gap

Despite extensive research in this field, several critical issues remain unresolved.

Generalized knowledge community findings may not apply to medical scenarios in which physicians’ consultation time is a significant cost [27]. Given the substantial offline consultation and clinical workload, physicians have limited time and energy to engage in online knowledge services, suggesting that the findings from generalized knowledge communities may not hold true in medical contexts. Second, there are 2 distinct mechanisms through which economic incentives impact free knowledge activities, and their effects may vary depending on the research context. Third, there is no consensus on the spillover effect of economic incentives, especially regarding the quality and diversity of contributions.

This study examined Haodf, a leading online medical platform in China, to explore how economic incentives influence physicians’ free knowledge activities using the PSM and multi-period DID methods.

## Methods

### Theoretical Foundation and Hypothesis Development

Motivation theory is a fundamental framework for analyzing user behavior patterns in online communities [28-30], categorizing user motivations as intrinsic and extrinsic [31]. Intrinsic motivation stems from the inherent satisfaction or enjoyment derived from engaging in specific behaviors, serving as a core driving force that prompts users to participate spontaneously. In contrast, extrinsic motivation is associated with external rewards or incentives that prompt users to undertake particular actions due to external stimuli. Existing research identifies 2 possible pivotal effects. First, overemphasizing or primarily relying on extrinsic incentives can trigger a “crowding-out effect,” in which extrinsic incentives undermine or even replace intrinsic motivation. Second, extrinsic incentives can trigger a “spillover effect” if they aim to enhance individuals’ perceived competence without compromising their sense of autonomy. That is to say, when extrinsic incentives are regarded as acknowledgments of individual effort and contribution, they are more likely to bolster intrinsic motivation rather than diminish it [29,31].

This study focused on online health knowledge contribution, distinguishing between free and paid health knowledge contributions. In this case, physicians who participate in free knowledge contributions are primarily driven by intrinsic motivation, which arises from their passion for knowledge sharing, professional achievement, and social responsibility. Conversely, physicians’ involvement in paid knowledge contributions is largely influenced by extrinsic motivation, namely, economic incentives. Notably, given the typically strong prosocial orientation of physicians, their behavior in sharing knowledge often stems from a robust intrinsic motivation. Thus, we believe that the rewards from paid contributions not only elevate their sense of achievement and self-confidence but also further enhance their intrinsic motivation, prompting them to engage more actively in unpaid knowledge contributions. Moreover, receiving economic incentives may prompt physicians to devote additional time and effort to free knowledge activities to avoid being perceived as solely motivated by economic incentives and establish a positive reputation [23]. Consequently, we hypothesize that, on online medical platforms, physicians’ engagement in paid knowledge activities will produce a spillover effect on their free knowledge activities rather than a crowding-out effect. This effect is manifested through a significant increase in the quantity of free knowledge contributions, notable improvements in their quality, and an expansion of content diversity. To evaluate this, this study developed measurement indexes for content contributions from 3 dimensions: quantity, quality, and diversity. Quantity directly measures the frequency of physicians’ participation in knowledge services, serving as the most fundamental evaluation metric. It reflects the physicians’ enthusiasm and level of engagement in knowledge contribution. Quality pertains to the professionalism and accuracy of the physicians’ knowledge service content, with high-quality services meeting readers’ medical needs. Diversity assesses the richness of the physicians’ knowledge services, evaluating whether they provide a wide range of content in various formats to address different issues, emphasizing variety and comprehensiveness.

The quantity of physicians’ free article contributions directly reflects the intensity of physicians’ online knowledge contributions, which is crucial for patients to evaluate physicians’ online performance. On the basis of this, this study proposed that participation in paid knowledge activities has a positive spillover effect on the quantity of free content contributions made by physicians (hypothesis 1).

As important as quantity, the quality of free articles is also vital for the sustainable development of online medical platforms. By writing high-quality articles and showcasing their profound medical knowledge and rich clinical experience, physicians can establish a professional image and win the trust and respect of patients. High-quality content can attract more user attention and participation, accumulate a good reputation and reputation for the platform, and lay a solid foundation for future sustainable development.

Whether physicians will improve the quality of free content when they receive economic incentives has not yet been conclusive. Some studies have found that small incentives have a negative impact on contribution quality [32], such as reducing the length of comments [33] and conveying one-sided emotions [33,34], which is due to the weakening of intrinsic motivation. However, from the perspective of reputation building, improving the quality of free content helps physicians establish a reputation for quality content services in the community, thus reducing patients’ uncertainty before purchasing content, which will have a positive impact on improving physicians’ paid services. On the basis of this, this study proposed that participation in paid knowledge activities has a positive spillover effect on the quality of free content contributions by physicians (hypothesis 2).

Diversity is the distribution of differences in certain attributes among members [35]. Diversity is important for community performance, such as user diversity promoting community learning [36] and improving content collaboration efficiency and outcomes [35]. Question-and-answer communities that encourage participants to answer more new questions have a good performance in answering questions, creating long-term value and improving difficulty resolution.

Diversity in online medical communities can be divided into media diversity and content diversity. Media diversity refers to the diversity of media adopted by physicians to spread medical knowledge. High media diversity indicates that physicians have adopted more ways to participate in knowledge contribution. Richer media are more likely to attract consumers’ attention and affect their evaluation of content [37]. The existence of economic incentives may encourage physicians to adopt more diverse means to present content to patients.

Content diversity refers to the differences in vocabulary involved in the creation of medical knowledge by physicians. These differences in vocabulary can affect the content richness of the physicians’ articles. When the content diversity of an article is high, the article may have richer and more comprehensive explanations [38]. Research has found that economic incentives increase the content diversity of online community discussions [22]. On the basis of this, this study proposed that participation in paid knowledge activities has a positive spillover effect on the diversity of free content contributions by physicians (hypothesis 3).

**Figure 2:**
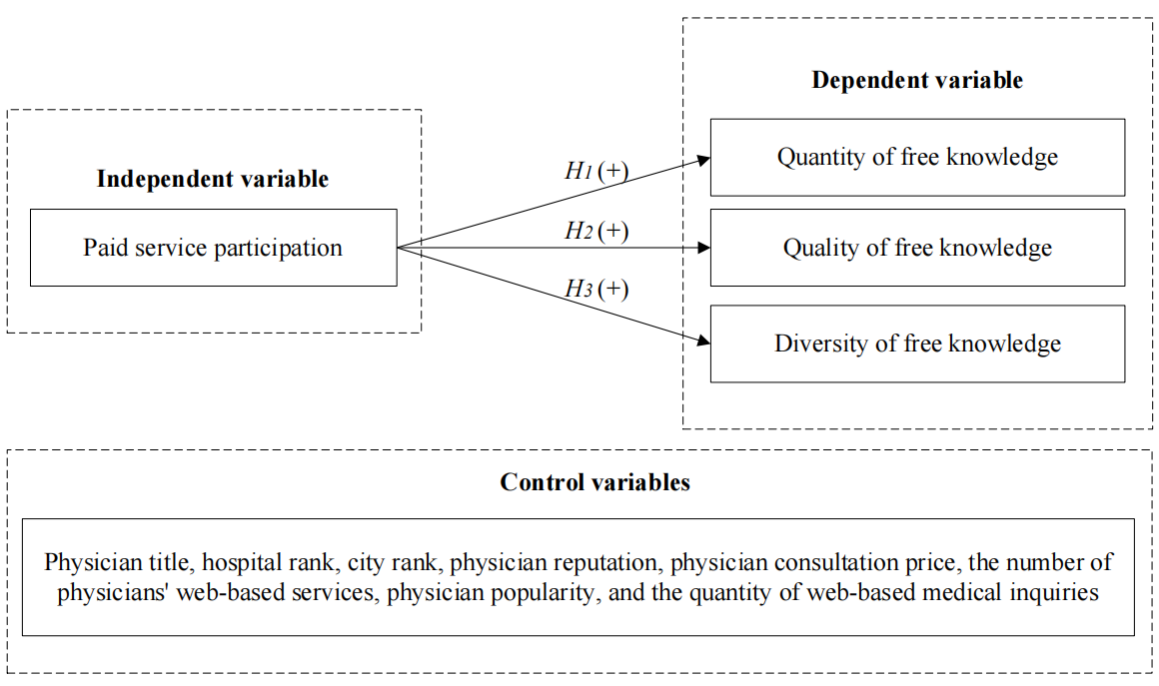
Research model of the spillover effects of the paid knowledge function. H: hypothesis.

The research model is shown in Figure 2.

### Sample Description

This study used the health knowledge column on the Haodf Online platform [39] as the data source. Haodf is one of the largest online medical websites in China, where many physicians provide diagnosis and treatment consultation services.

The paid medical knowledge service of the Haodf Online platform was introduced in 2016. The observation period for this study spanned 2016 to 2021. Due to the limited participation of physicians in knowledge activities in the short term, short intervals cannot effectively capture physicians’ knowledge activities and trends. Therefore, this study examined the impact of economic incentives on physicians’ knowledge activities over a relatively long period. Our time frame is divided into 6-month intervals [6], with the first interval covering data from January 1, 2016, to May 31, 2016, designated as spell 1. Each phase was set to 6 months to allow for a more accurate capture and analysis of the trends in physicians’ participation in online knowledge activities. Finally, the entire observation window is divided into 12 spells.

First, we comprehensively and systematically collected all the articles shown in the “Popular Science and Knowledge” section of the Haodf Online platform, totaling 63,151 articles. Subsequently, the authors of these articles, namely, the physicians, were the focus of our study. These physicians come from various departments covering the major medical specialties on the platform. After data cleaning, which involved removing physicians who had left the community and whose basic information could no longer be accessed, a total of 14,459 physicians were retained. Of these 14,459 physicians, 2070 (14.32%) participated in paid knowledge activities from 2016 to 2021, which constitutes the focal sample of this study. Using PSM with a 1:3 nearest neighbor matching technique, we matched physicians who participated in paid contributions with those who did not on a period-by-period basis, resulting in a comparison sample of 7453 physicians. Within this group of 7453 physicians, 2070 (27.77%) attempted to publish paid articles (although not necessarily in every period), whereas 5383 (72.23%) only published free articles. After conducting PSM on a period-by-period basis over the 6-year observation window, a total of 15,820 physician samples were obtained, including 6617 (41.83%) physician samples who participated in paid knowledge activities and 9203 (58.17%) physician samples who participated in free knowledge activities. The sample in this study is representative of the overall physician population in the Haodf Online medical community.

### Variable Definition

#### Dependent Variable

This study assessed the effectiveness of physicians’ contributions to free knowledge from 3 dimensions: quantity, quality, and diversity.

##### Quantity Dimension

The quantity dimension measures physicians’ contributions by the number of free articles published in the current period (*content_num*). Research indicates that economic incentives increase the number of content contributions in the community and cause widespread interest [22]. The community mainly takes the form of free articles without thresholds as knowledge contributions, and these free articles may be one of the important sources of physicians’ exposure in the community. When physicians participate in paid services, they may increase the number of free knowledge contributions to improve their exposure in the community and provide patients with a channel to evaluate the quality of the content.

##### Quality Dimension

The quality of free articles pertains to the professionalism and accuracy of the knowledge content produced by physicians, which can be evaluated from dimensions such as completeness, consistency, social consensus, accuracy, and usefulness [40]. Considering that the assessment of the accuracy of medical content requires the participation of medical professionals and the measurement of the usefulness of the information needs to take into account the needs and perceptions of patients, we omitted the 2 dimensions of accuracy and usefulness due to the insufficient support of secondhand data. This study selected the following 3 dimensions: completeness, consistency, and social consensus.

Completeness (*content_len*) is measured through the average of the number of characters in all free articles published by physicians in the current period, reflecting the detail and thoroughness of the information. The length of the information is directly related to the detail of the information. For example, research has found that the length of answers can best predict the quality of answers [41]. This study believes that, in the medical field, the length of answers is an important indicator, and the total number of words can be used to measure the completeness of answers.

Consistency (*simi_fuzz*) is measured through the similarity between the title and the full text [42], serving as an important indicator of information quality [43]. Whether the content is relevant to the topic directly affects the accurate transmission of medical content. This study used the *FuzzyWuzzy* package in Python (Python Software Foundation) to calculate the consistency score of the 2 groups of content. This toolkit is used for fuzzy string matching, and the difference between 2 sequences is calculated according to the edit distance algorithm.

Social consensus evaluates the common understanding among readers. This study considered whether the content contribution of physicians can promote communication and understanding from the perspective of suitability. Suitability is measured through the pragmatic features of free knowledge, which refer to language characteristics related to context, the speaker’s intention, the listener’s understanding, and sociocultural background in language use, reflecting the medical professionalism, logic, and readability of the article. From the perspective of the logic of content expression, due to economic incentives, physicians should enhance their logic to improve the experience of patients with reading free content. From the perspective of professional expression of content, due to economic incentives, physicians may enhance their professionalism to provide users with more professional medical content.

Pragmatic features were calculated using the TextMind software (Computational Cyber-Psychology Lab, Institute of Psychology, Chinese Academy of Sciences) [44,45], which categorizes words based on Linguistic Inquiry and Word Count. This method compares text words with dictionaries, calculating the proportion of different word categories in the text [46]. The relevant categories for this study included biological words, health words, functional words, and causal words. The proportion of biological (*bio*) and health (*health*) words reflects the medical professionalism of the content, improving the efficiency of physician-patient communication [45] and improving patient reading experiences [46]. The proportion of functional (*funct)* and causal (*cause*) words captures the language style and logical coherence of the content [47]. Textbox 1 shows the Linguistic Inquiry and Word Count word categories involved in this study and corresponding examples.

**Linguistic Inquiry and Word Count word categories in the Haodf online medical community.**

**Category and examples**

- *bio: dizzy, hug,* and *sweat*
- *health: insomnia, doctor,* and *exercise*
- *funct: maybe, many,* and *those*
- *cause: cause, make,* and *become*

##### Diversity Dimension

In online health communities, the diversity of knowledge services underscores the richness and comprehensiveness of knowledge. The diversity of physicians’ participation in knowledge services is primarily reflected in the presentation formats and the content they include. Physicians can use various media, such as images and text, to vividly present medical knowledge and cover a wide range of topics to ensure content richness. Therefore, the diversity dimension of physicians’ free knowledge contributions is measured through media diversity and content diversity. The number of media types and the information entropy of full text content were selected as the evaluation indexes for this dimension.

Media diversity (*tab_type*) refers to the variety of media types involved in the current free articles of physicians. The articles can take various forms, including popular science graphics, audio, and video. This study calculated the number of media types used in each physician’s article as a measure of media diversity.

Content diversity (*shanno_ent*) is measured through the information entropy of the current free articles of physicians. Information entropy represents the degree of uncertainty of the value, describing the amount of information content [48]. Higher entropy values indicate a higher degree of information complexity and greater information content, whereas lower entropy values indicate lower complexity and less information content. Information entropy has been applied in text sentiment classification [49], social influence analysis [50], comment usefulness analysis, personalized recommendation [51], and other fields. In this study, we preprocessed the free article content by cleaning and segmenting the text, counting the word frequency of each term, and then applying equations 1 and 2 to calculate the information entropy of each article. Finally, we calculated the average value of the information entropy of all free articles published by a physician in the current period as the content information entropy of the physician’s free knowledge contributions in the current month.

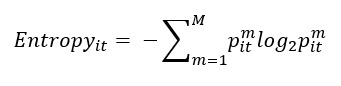

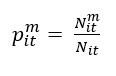





In the aforementioned formulas, *t* represents the *t*th period, *i*represents the *i*th physician, *m* represents the *m*th word, and *N_it_* represents the frequency of words in the free article published by physician *i* in the *t*th period.

#### Independent Variable

The main explanatory variable in this study was the participation of physicians in paid knowledge services, represented as *paid_service_it_*. It is a dummy variable to depict the participation of physicians in paid knowledge services. If the physician participated in paid knowledge services in the current period, the variable was coded as 1; otherwise, it was coded as 0.

#### Matching Variables

##### Overview

In PSM, matching variables are used to estimate the propensity score for each individual. These propensity scores measure the probability of an individual receiving the treatment or not (treatment or control). Matching variables typically include covariates related to the outcome variable, which help control for confounding factors, making the treatment and control groups as similar as possible on these variables. We synthesized previous research related to online health communities, identifying 3 primary components for matching variables: physician professional characteristics pertaining to their competency [52], online effort as an indicator of their experience and engagement [53], and online physician reviews reflecting patient evaluations [54].

##### Physician Professional Characteristics

Physician professional characteristics refer to their knowledge, skills, experience, and professional qualities in the medical field. Due to the variations in evaluating physicians’ titles across regions and hospitals, the professional capability dimension comprehensively considers physicians’ titles, the grade of the hospitals they work at [53], and the grade of the cities they work in [55].

Physician title (*title_add*) adds the clinical and academic titles of physicians. Physicians with higher titles, who are more likely to participate in online services, are better equipped to provide quality services [56]. Physician title is an important status capital, positively impacting physicians’ online participation performance [57,58].

Hospital rank (*hospital_rank*) ranks the hospitals where physicians work on a scale from 1 to 6, from low to high (first level=1, first class=2, second level=3, second class=4, third level=5, and third class=6). Hospital rank symbolizes the quality of the services provided by physicians and is another important offline capital for them [56].

City rank (*province_order*) is divided into 5 grades according to the development status of the province where the hospital is located and is recorded as high-quality area (5), medium-high–quality area (4), medium-quality area (3), medium-low–quality area (2), and element-lacking area (1). Cities with a higher economic development quality indicate that they have good resources, enabling physicians to gain better social advantages [58].

##### Online Effort

Online effort comprehensively measures physicians’ medical service performance on the platform, including the number of services offered, online consultation volumes. The website uses these metrics to reflect physicians’ activity and engagement levels [53]. This dimension also includes physicians’ consultation price, reflecting their value and experience.

The number of physicians’ online services (*service_num*) counts online services such as online diagnosis and treatment, physician team, private physician, and remote diagnosis. Considering the limited time and energy of physicians, they need to determine how to allocate their time to various tasks [59]. A wide service scope may lead to a decrease in the time that physicians allocate to free content creation, thus having a negative impact on the quantity, quality, and diversity of physicians’ free content.

The quantity of online medical inquiries (*inquiry_ln*) is measured using the logarithm of the quantity of online medical inquiries, reflecting the experience and participation of physicians in online services. The quantity of online medical inquiries is also a form of online knowledge contribution by physicians, which may have 2 directions of influence on their free knowledge contribution. On the one hand, online medical inquiries will occupy physicians’ time for online services, which could reduce the time available for them to contribute free content. On the other hand, physicians with more online inquiries are more active online and more likely to provide free knowledge for the community.

Physicians’ consultation price (*wenzhen_prince_ln*) is directly displayed on their home page and measures their assessment of their self-worth [56]. Physicians’ medical services are rewarded. For rational people’s consideration, physicians may pursue economic interests and put more effort into rewarding diagnosis and treatment services, which will have a crowding-out effect on free content contribution [26].

##### Online Physician Reviews

Online physician reviews refer to patient opinions [54], including physician popularity and reputation [60].

Physician popularity (*hot*) comes from the scores given by patients to physicians, ranging from 0 to 5. Physician popularity reflects the degree of recognition of physicians by patients and the personal brand influence of physicians and may have a positive impact on physicians’ online knowledge contribution. The reasons are as follows. First, the recommendation popularity to some extent conveys the degree of satisfaction of patients with physicians’ medical technology and communication level. Physicians with high popularity are more capable of providing a large amount of high-quality and rich free knowledge. Second, to maintain high scores from patients, physicians with high recommendation popularity are more motivated to give back to the community.

Physician reputation (*vote_each_month_ln*) is the number of patient votes received by physicians. It is the feedback and evaluation from patients of a physician’s service, indicating patient satisfaction. Physician reputation serves as an online status capital for physicians, motivating them to strengthen their unpaid content contributions to receive higher evaluations [56].

Table 1 shows the list of variables.

**Table 1:** Variable explanation.

Dimension and variable				Meaning		Explanation
**Dependent variables**						
	**Quantity**					
		content_num	Post number		Number of free articles posted by physicians	
	**Quality**					
		Completeness—content_len	Character count of free articles		The total number of characters in a free article posted by a physician	
		Consistency—simi_fuzz	Free article theme compliance		The similarity between the title and the content of free articles published by physicians (fuzzy set); the similarity score between the title and the content is calculated using the Python (Python Software Foundation) *FuzzyWuzzy* package	
		Social consensus—funct, social consensus—health, social consensus—bio, and social consensus—cause	Pragmatic feature		Use TextMind (Computational Cyber-Psychology Lab, Institute of Psychology, Chinese Academy of Sciences) to calculate the pragmatic features of free articles published by physicians; *funct* is the frequency of functional words in free articles published by physicians, *health* is the frequency of health words in free articles published by physicians, *bio* is the frequency of biological words in free articles published by physicians, and *cause* is the frequency of causal words in free articles published by physicians	
	**Diversity**					
		tab_type	Number of media types		Number of free articles with audio, video, and other multimedia content published by physicians	
		shanno_ent	Content information entropy		Equations 1 and 2 provide specific calculations	
**Independent variable**						
	paid_service_*it*_			The participation of physicians in paid knowledge services		A dummy variable—1 if participating and 0 otherwise
**Matching variables**						
	**Physician professional characteristics**					
		title_add	Physician title		The clinical and academic titles of physicians	
		hospital_rank	The rank of the hospital where the physician works		The grade of the hospitals where the physicians work, from low to high, is recorded as first level=1, first class=2, second level=3, second class=4, third level=5, and third class=6	
		province_order	The rank of the city where the physician works		Divided into 5 grades according to the development status of the province where the hospital is located and recorded as high-quality area=5, medium-high–quality area=4, medium-quality area=3, medium-low–quality area=2, and element-lacking area=1	
	**Online effort**					
		service_num	The number of physicians’ online services		The number of online services, such as online diagnosis and treatment, physician team, private physician, and remote diagnosis	
		inquiry_ln	The quantity of online medical inquiries		The logarithm of the quantity of online medical inquiries	
		wenzhen_price_ln	Physicians’ consultation price		The price of a single consultation provided by the physician	
	**Online physician reviews**					
		hot	Physician popularity		The scores given by patients to physicians, ranging from 0 to 5, with higher values indicating greater popularity	
		vote_each_month_ln	Physician reputation		The number of votes the physician has received from patients	

### Model Construction

This study used a quasi-experimental design that combines PSM with DID to study the impact of physicians’ participation in paid knowledge activities on their performance in free knowledge activities.

#### PSM Was Conducted to Reduce Selection Bias

PSM is a statistical method used to address data bias and confounding variables in observational studies, commonly used for causal inference. The presence of confounding variables can lead to an inaccurate estimation of treatment effects as certain characteristics may simultaneously influence both the likelihood of an individual receiving treatment and the study outcomes. This results in differences between the treatment and control groups that are not solely attributable to the treatment itself. Selection bias is also a significant concern. If the treatment and control groups differ substantially in baseline characteristics, these differences may arise from unobserved or difficult-to-quantify factors, leading to erroneous conclusions about the treatment effect. Directly comparing the 2 groups may, consequently, overestimate or underestimate the true impact of the treatment. The principle of PSM involves calculating the probability (propensity score) of each individual receiving a certain treatment (such as participating in a project or receiving a treatment) and then matching individuals in the treatment group with those in the control group who have similar propensity scores to reduce bias caused by confounding variables in the observed data, thus more accurately estimating the treatment effect. Common matching methods include one-to-one matching, one-to-many matching, one-to-many nearest neighbor matching, and match with replacement. One-to-one matching pairs each treatment group individual with a single closest control group individual, whereas one-to-many matching pairs each treatment group individual with multiple closest control group individuals. One-to-many nearest neighbor matching selects multiple closest control group individuals for each treatment group individual and chooses one for matching, improving matching accuracy. Match with replacement allows a control group individual to be matched with multiple treatment group individuals, enhancing flexibility. To improve the stability and accuracy of matched samples, this study used one-to-three nearest neighbor matching with replacement.

As physicians’ choice to participate in knowledge-based payment services is likely nonrandom and associated with factors such as offline capital and online service performance, sample selection bias may arise. To eliminate the influence of other characteristic differences between physicians participating in paid knowledge services and those not participating, this study initially used PSM to match samples and estimate the probability of physicians choosing paid knowledge services based on logit regression analysis [61].

Given a set of observable variables *Z*, the conditional probability that a physician *i* will enter the treatment group can be expressed as follows:

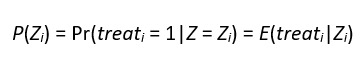



In this formula, *treat_i_* represents whether the physician has entered the treatment group. When *treat*=1, it indicates that the physician has been treated; otherwise, it is 0. The matching variables *Z* are selected from the following: physician title, hospital rank, city rank, physician reputation, physicians’ consultation price, number of physicians’ online services, physician popularity, and quantity of online medical inquiries.

After conducting PSM, the balance test is used to examine the effectiveness of the matching process. It checks whether the treatment and control groups have achieved a good balance in terms of covariates. The balance test is typically performed through statistical methods such as the 2-tailed *t* test and chi-square test, which compare the means, proportions, and other statistics of the covariates between the treatment and control groups before and after matching. If the differences in covariates between the 2 groups are not significant after matching, it is considered that a good balance has been achieved. Therefore, the balance test is one of the key indicators for evaluating the effectiveness of PSM results.

#### The Multi-Period DID Method Was Used to Estimate the Effect of Physicians’ Participation in Paid Knowledge Activities

The multi-period DID method is an econometric analysis technique developed from the traditional DID approach. It is used to evaluate the average causal effect of a policy or event on both the treatment and control groups across multiple time points. The principle of this method lies in comparing the changes within the treatment group before and after policy implementation (across multiple periods) with the changes within the control group during the same time frame. This allows for the estimation of the policy or event’s effect. This method effectively accounts for unobservable individual heterogeneity between the treatment and control groups as well as other time-varying trends, thus providing more accurate causal inferences.

As physicians joining paid knowledge services in this study may have occurred at different time points, to solve the endogeneity problem, we used the multi-period DID method for research, which takes into account the differences in the times when physicians entered the paid knowledge service. The DID method absorbs the unobserved differences between the treatment and control groups and eliminates the missing time trend related to physicians’ participation in paid knowledge services, which is widely used in policy evaluation. For the evaluation of multi-period policies, this study adopted the multi-period DID method [62], which can reduce the sample loss, include the data of physician participation in paid knowledge services in multiple periods, and make the results more general. In addition, physicians choose to participate in paid knowledge activities at different points in time, which poses multiple shocks to physicians’ online knowledge contribution. When the community launches paid knowledge activities, physicians may not immediately participate but may choose to participate after a period of observation. Therefore, the design model is as follows:

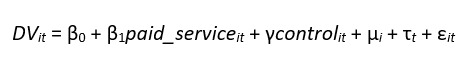



*i* represents the physician, *t* represents time, μ*_i_* is the individual fixed effect, τ*_t_* is the fixed effect of time, and *control_it_* represents other control variables. According to the basic idea of multi-time DID, the variable coefficient β_1_ reflects the change in physicians’ participation in free knowledge activities before and after the launch of paid knowledge activities, which is the main parameter to be estimated in this study.

### Ethical Considerations

This study did not require ethics approval as the authors only used publicly available content on the Haodf website and these data did not involve patient information.

## Results

### Results of PSM

In this study, we used the one-to-three nearest neighbor matching method with replacement for PSM [63]. In addition, in view of the time distribution of the articles published by physicians in each half-year span from 2016 to 2021, the year-by-year matching method (each spell as a *year*, representing half a year in our case) was adopted to find the matching control group for the treatment group in each spell. Taking the first period (ie, the first half of 2016) as an example, the predicted probability value of each physician’s engagement in free knowledge activities was first calculated in combination with the observable matching variables, and then 3 control group physicians who were not involved in paid knowledge activities were found for each physician participating in paid knowledge activities (the treatment group). After matching, physicians who were not successfully matched were excluded, resulting in 336 physicians in the treatment group for the first period. This method was consistently applied to subsequent periods, as shown in Table 2. After 12 periods, we obtained a total of 15,820 samples.

**Table 2:** Data situation of each period after propensity score matching.

Time dummy variable (spell_dummy)	Physicians, n	Participation in payment, n	Not participating in payment, n
1	576	240	336
2	612	263	349
3	978	409	569
4	1809	758	1051
5	2102	919	1183
6	1840	801	1039
7	2145	878	1267
8	1812	757	1055
9	1793	742	1051
10	893	346	547
11	734	290	444
12	526	214	312

**Figure 3:**
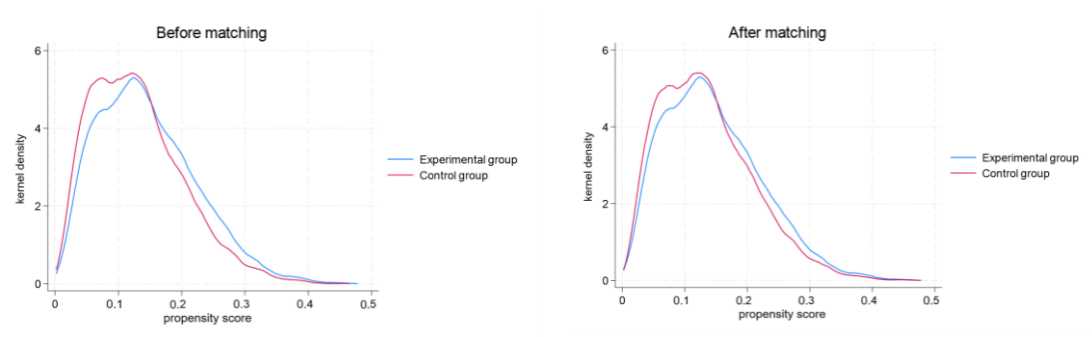
Kernel density plots before and after propensity score matching in phase 1.

Following PSM, we conducted a balance test using the 2-tailed *t* test method, and the results are presented in Table 3. The test results in Table 3 show that the absolute values of the bias of all matching variables were basically <5% after matching, indicating that the matching variables and matching methods selected in this study were reasonable. In addition, the *t* statistics after matching were not significant, suggesting no significant differences between the treatment and control groups after matching. Therefore, the samples obtained after matching ensure the randomness of sample processing and the reliability of the estimated results in this study. After PSM, the kernel density plots of the treatment group and the control group are closer to each other (as shown in Figure 3), indicating that the matching process successfully reduced the differences in covariates between the two groups, achieving better balance.

**Table 3:** Balance test results of propensity score matching for the first period.

Variable		Values, mean			Bias (%)		*t* test (*df*)		*P* value		V(T)^a^/V(C)^b^		Bias reduction (%)	
		Treatment	Control											
**hospital_rank^c^**														100
	Unmatched	5.713	5.687	2.4		0.26 (4075)		.80		0.83				
	Matched	5.712	5.712	0		–0.00 (574)		>.99		0.94				
**title_add^d^**														25.9
	Unmatched	5.092	5.078	0.6		0.07 (4075)		.94		0.99				
	Matched	5.074	5.064	0.5		0.04 (574)		.97		1.00				
**hot^e^**														74.6
	Unmatched	3.596	3.568	4.9		0.53 (4075)		.60		1.09				
	Matched	3.599	3.606	–1.2		–0.11 (574)		.91		0.97				
**vote_each_month_ln^f^**														–68.8
	Unmatched	2.668	2.623	3.3		0.35 (4075)		.73		0.81				
	Matched	2.677	2.602	5.5		0.49 (574)		.62		0.76				
**inquiry_ln^g^**														97.2
	Unmatched	7.836	7.720	8.5		0.94 (4075)		.35		1.13				
	Matched	7.869	7.866	0.2		0.02 (574)		.98		1.19				
**service_num^h^**														67.9
	Unmatched	1.933	1.837	8.4		0.90 (4075)		.37		0.93				
	Matched	1.945	1.914	2.7		0.24 (574)		.81		0.87				
**wenzhen_price_ln^i^**														–3.6
	Unmatched	3.419	3.375	3.4		0.37 (4075)		.71		1.31				
	Matched	3.440	3.395	3.5		0.32 (574)		.75		1.33				
**province_order^j^**														–76.4
	Unmatched	3.348	3.306	2.9		0.31 (4075)		.75		0.97				
	Matched	3.350	3.276	5.1		0.46 (574)		.65		0.97				

^a^V(T): variance in treatment group.

^c^hospital_rank: the rank of the hospital where the physician works.

^d^title_add: physician title.

^e^hot: physician popularity.

^f^vote_each_month_ln: physician reputation.

^g^inquiry_ln: the quantity of online medical inquiries.

^h^service_num: the number of physicians’ online services.

^i^wenzhen_price_ln: physicians’ consultation price.

^j^province_order: the rank of the city where the physician works.

bV(C): variance in control group.

After PSM, we conducted descriptive statistical analyses of the data (Table 4). The descriptive statistics revealed that the participation rate of physicians in paid knowledge services was 30.3% (4793/15,820) across all physician-period combinations. The median of 0 (IQR 0.000-1.000) indicates that the overall sample had a low participation rate in paid knowledge services. Furthermore, the data distribution of physicians within the sample shows significant variation in the dimensions of quantity, quality, and diversity of knowledge services, indicating potential areas for further improvement.

**Table 4:** Summary statistics.

Variable name			Observations, n		Values, mean (SD)		Values, median (IQR; range)
**Independent variable**							
	paid_service^a^	15,820		0.303 (0.460)		0.000 (0.000-1.000; 0.000-1.000)	
**Dependent variable**							
	content_num^b^	15,820		8.841 (33.272)		3.000 (1.000-7.000; 1.000-2029.000)	
	content_len^c^	15,820		1201.667 (1383.669)		925.535 (500.000-1493.250; 0.000-43,188.000)	
	simi_fuzz^d^	15,820		3.332 (5.895)		2.000 (1.000-3.438; 0.000-100.000)	
	funct^e^	15,820		0.250 (0.099)		0.273 (0.221-0.312; 0.000-0.625)	
	health^f^	15,820		0.054 (0.037)		0.058 (0.024-0.078; 0.000-0.750)	
	bio^g^	15,820		0.078 (0.051)		0.089 (0.041-0.113; 0.000-1.000)	
	cause^h^	15,820		0.019 (0.015)		0.019 (0.002-0.029; 0.000-0.222)	
	tab_type^i^	15,820		1.046 (0.236)		1.000 (1.000-1.000; 1.000-4.000)	
	shanno_ent^j^	15,532		7.288 (1.549)		7.673 (7.303-7.958; 0.000-9.397)	
**Matching variables**							
	title_add^k^	15,820		4.504 (2.144)		4.000 (3.000-6.000; 0.000-8.000)	
	hospital_rank^l^	15,820		5.570 (1.334)		6.000 (6.000-6.000; 0.000-6.000)	
	province_order^m^	15,820		3.351 (1.490)		4.000 (2.000-5.000; 0.000-5.000)	
	vote_each_month_ln^n^	15,820		1.802 (1.424)		1.792 (0.693-2.944; 0.000-6.842)	
	wenzhen_price_ln^o^	15,820		3.249 (1.218)		3.367 (2.398-4.111; 0.000-7.314)	
	service_num^p^	15,820		1.788 (1.136)		2.000 (1.000-3.000; 0.000-4.000)	
	hot^q^	15,820		3.507 (0.545)		3.400 (3.100-3.800; 0.000-5.000)	
	inquiry_ln^r^	15,820		7.235 (1.523)		7.412 (6.371-8.286; 0.000-11.752)	

^a^paid_service: the participation of physicians in paid knowledge services.

^b^content_num: post number.

^c^content_len: character count of free articles.

^d^simi_fuzz: free article theme compliance.

^e^funct: the frequency of functional words in free articles published by physicians.

^f^health: the frequency of health words in free articles published by physicians.

^g^bio: the frequency of biological words in free articles published by physicians.

^h^cause: the frequency of causal words in free articles published by physicians.

^i^tab_type: number of media types.

^j^shanno_ent: content information entropy.

^k^title_add: physician title.

^l^hospital_rank: the rank of the hospital where the physician works.

^m^province_order: the rank of the city where the physician works.

^n^vote_each_month_ln: physician reputation.

^o^wenzhen_price_ln: physicians’ consultation price.

^p^service_num: the number of physicians’ online services.

^q^hot: physician popularity.

^r^inquiry_ln: the quantity of online medical inquiries.

### Results and Analysis of the Multi-Period DID Estimation

On the basis of the PSM estimation, this study identified a new control group with similar characteristics to those of the treatment group. The treatment and control groups encompassed a total of 7453 physicians and 15,820 samples. For these matched samples, we constructed dependent variables from the dimensions of quantity, quality, and diversity. The multi-period DID method was used to examine the impact of physicians’ engagement in paid knowledge activities on their participation in free knowledge activities. The detailed estimation results are shown in Tables 5 and 6. Table 5 illustrates the pure treatment effects on the dimensions of quantity, quality, and diversity of various dependent variables derived from a multi-period DID analysis after eliminating confounding factors. To validate the robustness of these findings, Table 6 incorporates additional regression analyses with control variables. The results of Table 6 are largely consistent with those of Table 5, thereby strengthening the reliability and validity of the treatment effects.

**Table 5:** Estimation results of difference in differences (no controls).

	Quantity—content_num^a^ (model 1)	Quality							Diversity	
		content_len^b^ (model 2)	simi_fuzz^c^ (model 3)	funct^d^ (model 4)	health^e^ (model 5)	bio^f^ (model 6)	cause^g^ (model 7)	tab_type^h^ (model 8)		shanno_ent^i^ (model 9)

paid_service^j^, estimated value (*P* value)	5.071 (<.001)	106.100 (.01)	–1.121 (<.001)	0.006 (.001)	0.000 (.76)	0.001 (.55)	0.002 (<.001)	0.004 (.02)		0.065 (.007)
Observations, N	15,820	15,820	15,820	15,820	15,820	15,820	15,820	15,820		15,532
*R* ^2^	0.005	0.006	0.010	0.054	0.038	0.049	0.015	0.033		0.075
Adjusted *R*^2^	0.004	0.005	0.010	0.053	0.037	0.048	0.014	0.032		0.074

^a^content_num: post number.

^b^content_len: character count of free articles.

^c^simi_fuzz: free article theme compliance.

^d^funct: the frequency of functional words in free articles published by physicians.

^e^health: the frequency of health words in free articles published by physicians.

^f^bio: the frequency of biological words in free articles published by physicians.

^g^cause: the frequency of causal words in free articles published by physicians.

^h^tab_type: number of media types.

^i^shanno_ent: content information entropy.

^j^paid_service: the participation of physicians in paid knowledge services.

**Table 6:** Estimation results of difference in differences (with controls).

	Quantity—content_num^a^ (model 10)	Quality							Diversity	
		content_len^b^ (model 11)	simi_fuzz^c^ (model 12)	funct^d^ (model 13)	health^e^ (model 14)	bio^f^ (model 15)	cause^g^ (model 16)	tab_type^h^ (model 17)		shanno_ent^i^ (model 18)
paid_service^j^, estimated value (*P* value)	4.731 (<.001)	108.000 (.009)	–1.155 (<.001)	0.006 (.001)	0.001 (.64)	0.001 (.45)	0.002 (<.001)	0.003 (.09)		0.066 (.006)
hospital_rank^k^, estimated value (*P* value)	—^l^	—	—	—	—	—	—	—		—
title_add^m^, estimated value (*P* value)	–1.321 (.60)	102.900 (.72)	–3.763 (.15)	–0.067 (.11)	–0.007 (.64)	–0.037 (.001)	–0.003 (.60)	–0.023 (.13)		–1.006 (.37)
hot^n^, estimated value (*P* value)	76.450 (.55)	1659.900 (.92)	120.800 (.23)	4.517 (.04)	0.355 (.60)	1.886 (.003)	0.225 (.50)	1.998 (.25)		—
vote_each_month_ln^o^, estimated value (*P* value)	2.322 (<.001)	–13.140 (.39)	0.237 (.001)	–0.001 (.26)	–0.001 (.004)	–0.001 (.02)	0.000 (.78)	0.007^o^ (.04)		–0.005 (.82)
inquiry_ln^p^, estimated value (*P* value)	–256.800 (.72)	113,611.900 (.24)	82.680 (.74)	6.442 (.64)	5.590 (.09)	3.299 (.43)	0.816 (.72)	–15.460 (.26)		104.400 (.36)
service_num^q^, estimated value (*P* value)	—	—	—	—	—	—	—	—		—
wenzhen_price_ln^r^, estimated value (*P* value)	7.399 (.02)	2078.500 (<.001)	–0.756 (.37)	0.029 (.34)	–0.220 (<.001)	–0.214^t^ (<.001)	0.094^t^ (<.001)	0.055 (.13)		–0.164 (.56)
province_order^s^, estimated value (*P* value)	—	—	—	—	—	—	—	—		—
Observations, N	15,820	15,820	15,820	15,820	15,820	15,820	15,820	15,820		15,532
*R* ^2^	0.008	0.006	0.012	0.056	0.040	0.051	0.016	0.034		0.075
Adjusted *R*^2^	0.007	0.005	0.011	0.055	0.039	0.050	0.014	0.033		0.074

^a^content_num: post number.

^b^content_len: character count of free articles.

^c^simi_fuzz: free article theme compliance.

^d^funct: the frequency of functional words in free articles published by physicians.

^e^health: the frequency of health words in free articles published by physicians.

^f^bio: the frequency of biological words in free articles published by physicians.

^g^cause: the frequency of causal words in free articles published by physicians.

^h^tab_type: number of media types.

^i^shanno_ent: content information entropy.

^j^paid_service: the participation of physicians in paid knowledge services.

^k^hospital_rank: the rank of the hospital where the physician works.

^l^Not applicable.

^m^title_add: physician title.

^n^hot: physician popularity.

^o^vote_each_month_ln: physician reputation.

^p^inquiry_ln: the quantity of online medical inquiries.

^q^service_num: the number of physicians’ online services.

^r^wenzhen_price_ln: physicians’ consultation price.

^s^province_order: the rank of the city where the physician works.

In the quantity dimension, model 10 shows that physicians’ participation in paid knowledge activities led to a significant 473.1% increase in the number of free articles (*P*<.001). This substantial increase aligns with the findings of previous research [1,6]. The consumer’s knowledge payment behavior helps to promote the contribution of free knowledge.

According to Table 6, in the dimension of quality, the participation in paid knowledge activities significantly promoted physicians’ performance in terms of content length, frequency of functional words, and frequency of causal words. The result of model 11 demonstrates that physicians’ participation in paid knowledge activities had a positive spillover effect on the length of free articles (108%; *P*=.009), which is inconsistent with the conclusion that economic incentives do not affect the quality of free services in previous study [6,22]. This may be due to physicians’ desire to enhance the online reputation of their free content and establish a good online reputation, thereby attracting more traffic to their paid services. The result of model 12 indicates that physicians’ participation in paid knowledge activities had a negative effect on the consistency between titles and content in free articles, with a reduction of 115.5% (*P*<.001). This is inconsistent with our initial hypothesis. Further investigation on examples of lower similarity scores is shown in Table S1 in Multimedia Appendix 1. It indicates that physicians often use eye-catching expressions and summaries in titles to engage readers, which may lead to less alignment between titles and content. We further conducted supplementary analyses (Table S2 in Multimedia Appendix 1). First, a *t* test comparing the consistency between titles and content in free versus paid knowledge after physicians engaged in paid knowledge activities was conducted. The results revealed that, within 12 months of initiating paid activities, the consistency between titles and content in free knowledge services remained significantly higher than in paid knowledge services. This may be because paid knowledge products require more attention-grabbing and creative titles to enhance click-through and conversion rates, thereby reducing consistency between titles and content. In the short term, physicians might also adjust the titles of their free knowledge services, resulting in a decrease in title-content consistency for free services after adopting the paid service, although consistency remained higher than for paid services. Second, over the 12 months following the initiation of the paid service, consistency between titles and content in both free and paid knowledge services gradually improved. This trend may be attributed to physicians accumulating more experience in providing knowledge services over time, enabling them to better balance creativity and content, thereby progressively enhancing the consistency between titles and content. The result of model 13 shows that physicians’ participation in paid knowledge activities had a positive effect on the frequency of functional words in free articles, which increased by 0.6% (*P*=.001). Physicians adopt different language styles in the expression of free content, which may be a differentiation strategy to distinguish paid content from free content. Model 14 indicates that there was no significant effect on the frequency of health-related words in free articles. Similarly, the result of model 15 shows no significant effect on the frequency of biological words. The changes in health word and biological word frequency before and after receiving the economic incentive were not significant, which shows that physicians did not pursue the output of professional content, possibly to maintain the universal positioning of free content. The result of model 16 reveals that physicians’ participation in paid knowledge activities positively impacted the frequency of causal words in free articles, which increased by 0.2% (*P*<.001). Physicians increased the frequency of causal words, which may be to narrate medical content with greater logic.

In the dimension of diversity, participation in paid knowledge activities had a significant impact on the content information entropy of free knowledge services. The result of model 8 indicates that physicians’ participation in paid knowledge activities had a positive impact on the media diversity of free articles, but the significance level was relatively low (*P*=.02). However, model 17 shows that physicians’ participation in paid knowledge activities had no significant impact on the media diversity of free articles. This discrepancy may arise from path dependency in physicians’ choice of media as they tended to continue using previously used media, leading to minimal variation in media use before and after engaging in paid knowledge activities. The result of model 18 indicates that physicians’ participation in paid knowledge activities had a positive effect on the content information entropy of free articles, which increased by 6.6% (*P*=.006). This finding aligns with the conclusion by Wang et al [6]. Economic incentives encourage physicians to produce more diverse and rich content to the community, contributing to its vitality and development.

Overall, the results demonstrate that the physicians’ participation in paid knowledge activities significantly enhanced their engagement in free knowledge activities. In terms of quantity, participation in paid knowledge activities substantially increased the number of free articles published by physicians. In terms of quality, paid knowledge participation had a significant positive impact on the length, function word frequency, and causal word frequency of free articles while negatively affecting the consistency between title and content. It had no significant impact on the frequency of health-related and biological words. In terms of diversity, paid knowledge participation significantly improved the content information entropy of free articles but did not notably affect media diversity.

## Discussion

### Principal Findings

The research question addressed in this study was the impact of economic incentives on physicians’ free knowledge contribution in online medical communities. To elucidate this issue, this study used a quasi-experimental method combining PSM with multi-period DID to verify the spillover effect of knowledge payment activities on free knowledge contributions across dimensions of quantity, quality, and diversity. The sample selection bias problem was controlled through PSM. The findings revealed that economic incentives exerted spillover effects on physicians’ free knowledge contributions, affecting all 3 dimensions. Specifically, economic incentives had a positive spillover effect on the quantity, length, and frequency of functional words; frequency of causal words; and content information entropy of free articles while negatively impacting the consistency between titles and content of free articles.

### Theoretical Implications

Theoretically, this study enriches understanding of economic incentives within the realm of online health care and explores physicians’ online knowledge contributions from a multidimensional perspective. The contributions of this study are as follows. First, this study comprehensively explored the impact of economic incentives on physicians’ free online medical knowledge contributions, enriching the literature on digital health care and knowledge contribution. Previous studies have mostly focused on nonspecific fields or broad knowledge-sharing platforms [5], with few investigating the motivational mechanisms for knowledge contribution in the specific context of health care. By introducing motivation theory, this study validated the spillover effect of paid knowledge contributions on free knowledge contributions by physicians, providing a theoretical basis and decision support for the sustainable and orderly development of paid knowledge services in online medical communities. Second, this study constructed a comprehensive, 3D knowledge evaluation framework. Previous research has primarily focused on the quantity and quality of knowledge [31,34]. This study further introduced the important dimension of *diversity*. In terms of measurement methods, earlier research has largely relied on structured data. In contrast, this study used text-mining techniques to extract information related to knowledge quality, such as the consistency between titles and content, function words, health-related vocabulary, physiological process terms, causal words, and entropy values related to knowledge diversity. Third, this study used a year-by-year PSM and a multi-period DID method to scientifically evaluate the impact of economic incentive models on the platform. By constructing a counterfactual framework, the PSM method was used to introduce physicians who did not participate in paid knowledge contributions as a control group, scientifically assessing the impact of participation in paid knowledge contributions on physicians’ free knowledge activities. Simultaneously, the year-by-year DID reduced the selection bias of physicians who published paid content at different time points and, in combination with text-mining methods, explored the impact of economic incentives on physicians’ online knowledge participation on the platform.

### Practical Implications

This study also has certain practical implications. First, for the platform, providing economic incentives to physicians can promote the overall development of the platform’s knowledge content ecosystem. The introduction of economic incentives can be a feasible business model for knowledge platforms to stimulate users’ contributions in paid and related unpaid activities. Currently, some free medical knowledge–sharing platforms, such as Dingxiangyuan and Kuaishou Health, have introduced paid knowledge through paid courses or short videos. This study holds significant reference value and strategic guidance for these platforms. In addition, this study also provides guidance for nonpaid medical knowledge platforms such as Chunyu Doctor and Baidu Health, offering valuable insights and strategic direction for introducing brand-new paid knowledge models. For paid knowledge platforms in other countries that operate on a membership basis or support one-time payments, such as medical-related communities on Patreon and medical-related modules on Substack, the research framework and related conclusions of this study can also provide valuable insights and references. Second, regarding physicians, they need to maintain their attention and investment in free knowledge activities and effectively manage them as a traffic entrance for paid users. Third, for patients, economic incentives have spillover effects on their free experience, helping them enjoy more, better, and more diverse knowledge articles for free.

### Limitations

There are still some limitations to this study, which can be studied in depth in the future. First, the observation period was relatively short. This study only chose half a year as an observation period for the release of paid articles, primarily focusing on the medium- to long-term economic incentive effects. In the future, the observation period can be extended to study the long-term changes in spillover effects and further explore the impact of economic incentives on physicians’ long-term professional development and knowledge-sharing behaviors. Second, in addition to economic incentives, the influence of noneconomic incentives (such as reputation) on physicians’ online knowledge service behaviors is a significant topic for future research. In the future, further in-depth exploration can be conducted on the noneconomic incentive measures of the platform and their impact on physicians’ online knowledge service behaviors. Third, in terms of the presentation format of knowledge content, only the textual form of products was studied. In the future, we can use speech transcription–related tools to extract information from videos, speech, and other forms of products and fully consider all types of articles. Fourth, this study was based on an online medical platform in China. Given that cultural backgrounds would profoundly influence individuals’ values, motivational systems, and decision-making processes, the conclusions drawn from Chinese platforms may offer only limited applicability, and there are some limitations in applying these findings to other countries and regions. Future research should fully consider the influence of cultural backgrounds and conduct more in-depth studies in this regard.

## Appendix

Multimedia Appendix 1 Further analysis of the consistency between titles and content.

## Abbreviations

DID: difference in differences
PSM: propensity score matching

## Data Availability

The datasets generated during and analyzed during this study are available from the corresponding author on reasonable request.
