# Supplementary material for: Spillover Effects of Paid Functions on Physicians’ Unpaid Knowledge Activities: Quasi-Experimental Approach
Source: J Med Internet Res. 2024 Dec 10;26:e58688. doi: 10.2196/58688 (PMC11668984; doi:10.2196/58688)
Supplement: Multimedia Appendix 1 [file jmir_v26i1e58688_app1.docx]

**Appendix 1: Further Analysis on the Consistency Between Titles and Content**

Table A1 shows some examples of lower similarity scores. In most cases, the purpose of the title of a doctor's popular science article is to hint at the content of the article and attract readers to read it. However, there are also two special cases: artistic expression in the title and different focuses between the title and the full text, which lead to differences in keywords between the title and the content, resulting in lower similarity scores. Such examples include: using interrogative titles to introduce and lead into the full text, while the full text expands on the explanation and supplements more details; using artistic techniques such as poetic allusion and anthropomorphic expression in the title; using network buzzwords and colloquial expressions in the title; using characters and plots from movies and TV shows in the title; focusing on the significance of understanding the treatment of the disease in the title, while the full text focuses on explaining the specific manifestations and clinical treatment methods of the disease; and briefly summarizing the title, while the full text provides a detailed explanation of the disease. Therefore, there are indeed differences in the consistency of keywords between the title and the content.

**Table A1. Examples of low Consistency of Title and Content scores**

| Reasons | Title | Keywords of content |
| --- | --- | --- |
| Question-type titles | Does Consuming More Than 1 Egg per Day Increase the Risk of Cardiovascular Disease? | 'Egg', 'Intake', 'Cardiovascular', 'Cholesterol', 'Disease', '95%', 'Risk', 'Intake', 'HR', 'CI' |
|  | 10 types of topical anti-itch medicines, will you choose? | 'Itchy', 'External', 'Itch-relieving', 'Cream', 'Chronic', 'Dermatitis', 'Effect', 'Inhibitor', 'Calcium', 'Nodular' |
|  | Is it true that many Chinese people have lactose intolerance? | 'lactose', 'drink milk', 'tolerance', 'intake', 'symptoms', 'most', 'can be up to', '90%', 'intolerance', '200' |
|  | What should I do if my teeth turn yellow or black? | 'Teeth', 'Tetracycline tooth', 'Color', 'Dental fluorosis', 'Abnormal', 'Treatment', 'Exogenous', 'Prevention', 'Dental calculus', 'Dental veneer' |
| Titles using poetic and anthropomorphic expressions | Tension Headaches: Why Do They Happen at the Top? | 'Headache', 'Tension', 'Bilateral', 'Attack', 'Pain', 'Treatment', 'Relaxation Therapy', 'Patient', 'Aggravate', 'Muscle' |
|  | How to Correctly Understand Precocious puberty: In fact, you don't understand my heart | 'Precocious puberty', 'Child', 'Development', 'GnRHa', 'Treatment', 'Height', 'Patient', '12', 'Tumor', 'Gonadotropin-releasing hormone' |
| Titles using colloquial expressions in titles | Can a herniated disc be dissolved? Yes! With collagenase yyds! | 'Collagenase', 'Treatment', 'Disc', 'Dissolution', 'Conservative', 'Surgery', 'Nucleolysis', 'Absorption', 'Epidural', 'Discectomy' |
| Titles using movie and TV character plots | Yonglian kills himself - asthma | 'Asthma', 'Treatment', 'Childhood asthma', 'Attack', 'Cough', 'Airway', 'Bronchial', 'ICS', 'LABA', 'Function' |
| Different emphasis on content in titles | Rib Cartilage - Reshaping Beautiful Features | 'Carpopharynx', 'Ear surgery', 'Autologous', 'Rhinoplasty', 'Ribs', 'Auricle', 'Surgery', 'Can', 'Cartilage', 'Post-surgery' |
|  | Meniere's Disease: Time to Destigmatize | 'Meniere', 'Vertigo', 'Diagnosis', 'Vestibular', 'Hearing', 'Patient', 'Symptoms', 'Attack', 'Ear swelling', 'Duration' |
| The title briefly summarizes, and the content is detailed. | The correct treatment method for allergic rhinitis | 'Allergic', 'Rhinitis', 'Allergen', 'Nasal', 'Treatment', 'Allergy', 'Symptoms', 'Corticosteroid', 'Drug', 'Exposure' |
|  | 7 Answers Everyone Needs to Know After Changchun Changsheng Vaccine Incident | 'Vaccine', 'DPT', 'Inoculation', 'Batch', 'Problem', 'Involved', 'Parents', 'Children', 'Prevention', 'Pertussis' ' |

Table A2 illustrates the changes in the consistency of title and content for both paid and free knowledge services over the 12 months following doctors' participation in paid knowledge activities. Additionally, a T-test was conducted to examine the consistency of titles and content for each period of paid and free knowledge services.

**Table A2.** Changes in the Consistency of Title and Content of Doctors' Knowledge after Adopting Paid Knowledge Services

| Variable | ttest_statistic | ttest_pvalue | mean_paid | mean_free | Time after participating in paid knowledge activities |
| --- | --- | --- | --- | --- | --- |
| simi_fuzz | -6.538 | 0.000 | 1.815 | 2.726 | 1 month |
|  | -3.611 | 0.000 | 1.853 | 2.725 | 3 months |
|  | -4.087 | 0.000 | 1.801 | 3.169 | 6 months |
|  | -3.506 | 0.000 | 1.673 | 3.128 | 9 months |
|  | -1.026 | 0.305 | 3.192 | 4.154 | 12 months |
